# A highly efficient method for genomic deletion across diverse lengths in thermophilic *Parageobacillus thermoglucosidasius*

**DOI:** 10.1016/j.synbio.2024.05.009

**Published:** 2024-05-17

**Authors:** Zhiheng Yang, Bixiao Li, Ruihong Bu, Zhengduo Wang, Zhenguo Xin, Zilong Li, Lixin Zhang, Weishan Wang

**Affiliations:** a State Key Laboratory of Bioreactor Engineering, School of Biotechnology, East China University of Science and Technology (ECUST), 200237, Shanghai, China; b Beijing Key Laboratory of Photoelectronic/Electrophotonic Conversion Materials, Key Laboratory of Cluster Science, Ministry of Education, Frontiers Science Center for High Energy Material, Advanced Research Institute of Multidisciplinary Science, School of Chemistry and Chemical Engineering, Beijing Institute of Technology, Beijing, 100081, China; c State Key Laboratory of Microbial Resources, Institute of Microbiology, Chinese Academy of Sciences, Beijing, 100101, China

**Keywords:** Type I CRISPR system, Thermostable NHEJ enzymes, Thermophile, Long-range genomic deletions

## Abstract

*Parageobacillus thermoglucosidasius* is emerging as a highly promising thermophilic organism for metabolic engineering. The utilization of CRISPR-Cas technologies has facilitated programmable genetic manipulation in *P. thermoglucosidasius*. However, the absence of thermostable NHEJ enzymes limited the capability of the endogenous type I CRISPR-Cas system to generate a variety of extensive genomic deletions. Here, two thermophilic NHEJ enzymes were identified and combined with the endogenous type I CRISPR-Cas system to develop a genetic manipulation tool that can achieve long-range genomic deletion across various lengths. By optimizing this tool—through adjusting the expression level of NHEJ enzymes and leveraging our discovery of a negative correlation between GC content of the guide RNA (gRNA) and deletion efficacy—we streamlined a comprehensive gRNA selection manual for whole-genome editing, achieving a 100 % success rate in randomly selecting gRNAs. Notably, using just one gRNA, we achieved genomic deletions spanning diverse length, exceeding 200 kilobases. This tool will facilitate the genomic manipulation of *P. thermoglucosidasius* for both fundamental research and applied engineering studies, further unlocking its potential as a thermophilic cell factory.

## 1 Introduction

Biomanufacturing via microbial cell factories has revolutionized the production of numerous chemicals, offering an eco-friendly alternative to traditional fossil fuel–based processes [1]. However, the economic viability of biomanufacturing often falls short when compared to conventional manufacturing methods. One primary factors contributing to this disparity is the susceptibility of mesophile strains to contamination, which hampers their viability in production processes [2]. Consequently, thermophilic strains have garnered attention as promising candidates for contamination-resistant bioprocessing [3]. By harnessing thermophile strains in fermentation, not only can contamination risks be reduced at elevated temperatures, but also feedstock conversion rates can be improved, bioreactor cooling costs can be minimized, and viscosity levels during fermentation can be decreased [4]. Among these thermophilic candidates, *Parageobacillus thermoglucosidasius* exhibits great application potential due to its versatility in fermenting a broad range of substates into valuable products, spanning from chemicals to alternative energy sources and enzymes [[5], [6], [7], [8], [9], [10], [11]]. To promote efficiently engineering of this promising thermophilic strain, an important prerequisite is to establish genome manipulation tools.

CRISPR arrays and their associated Cas (CRISPR-associated) proteins endow prokaryotes with adaptive and heritable immunity [12]. The type I CRISPR-Cas system is the most prevalent in nature [13]. This scenario has facilitated genetic editing of non-model microorganisms by harnessing their native CRISPR-Cas3 systems [[14], [15], [16], [17], [18], [19]]. We have previously developed efficient tools for genetic manipulation in *P. thermoglucosidasius* based on the endogenous type I CRISPR-Cas system, enabling capabilities in genetic editing, gene regulation, CRISPRi (CRISPR interference) library, and the dynamically controlled supercompetent cells [20]. Although exhibiting excellent efficiency, we found that this type I CRISPR systems failed to generate long-range genomic deletion in *P. thermoglucosidasius*. This results differ from reports that type I CRISPR system could generate large genomic deletions of varying lengths due to the processive degradation capability of the Cas3 nuclease-helicase [[21], [22], [23]].

To discover the underlying reason, we noted that these achievements of large genomic deletions using type I CRISPR system were only realized in human cells or bacteria with an endogenous nonhomologous ending joining (NHEJ) or alternative-end joining (AJ) repair system [[21], [22], [23]]. However, *P*. *thermoglucosidasius* lacks such NHEJ or AJ repair systems. Thus, we concluded that the absence of efficient ligation system is the main obstacle to successful long-range genomic deletion when using the type I CRISPR system in *P*. *thermoglucosidasius*. Compared to the AJ repair system, the NHEJ repair system is currently more clearly studied in bacteria [24]. In generally, the NHEJ process in bacteria requires only the Ku protein and ligase D (LigD) [24,25]. Additionally, in the absence of LigD, Ligase C (LigC) along with the Ku protein have been demonstrated to repair broken genomes in mycobacteria [26]. The Ku protein binds to DNA ends, recruiting LigD, which then processes and ligates the DNA ends [27]. Although the mechanism of NHEJ has been elucidated, no active NHEJ enzymes from thermophilic organisms have been identified until now [24]. This gap underscores the urgent need to identify thermostable NHEJ enzymes. Such enzymes could achieve highly efficient ligation following the extensive genomic deletions created around the target sites in *P. thermoglucosidasius.*

In this study, we identified two thermostable NHEJ enzymes capable of ligating genomic breaks of varying lengths created by the endogenous type I CRISPR-Cas system. We next developed an autoregulated xylose autoinduction strategy in a glucose-xylose medium to delay gRNA transcription initiation, resulting in enhanced editing efficiency conveniently. Additionally, we observed that gRNAs with low GC content significantly improved deletion efficiency. Building upon these findings, we developed a comprehensive whole-genome gRNA selection manual using a Python script. Notably, all seven gRNAs selected from this manual successfully achieved editing outcomes, including one gRNA enabling deletions exceeding 200 kilobases. Overall, leveraging the endogenous type I CRISPR-Cas system coupled with NHEJ enzymes, we have devised a genetic manipulation tool facilitating efficient large fragment genomic deletions in *P. thermoglucosidasius*, which will greatly unlock the potential of *P*. *thermoglucosidasius* for industrial applications.

## 2 Methods

### 2.1 Bacterial strains and growth conditions

The bacterial strains utilized in this study are detailed in Table S1. *P. thermoglucosidasius* NCIMB 11955 was acquired from the China General Microbiological Culture Collection Center (CGMCC). *Escherichia coli* JM109 was regularly grown at 37 °C in Luria-Bertani (LB) medium. *P. thermoglucosidasius* cultures were maintained in modified LB (mLB) medium, which is LB medium supplemented with 0.59 mM MgSO_4_·7H_2_O, 0.91 mM CaCl_2_·2H_2_O, and 0.04 mM FeSO_4_·7H_2_O, or on TSA (Oxoid™, CM0131) agar plates. For fermenting *P. thermoglucosidasius*, a 2 × YT medium supplemented with 10 g/L glucose was used in 250 ml flasks, agitated at 250 rpm, at a temperature of 60 °C. Antibiotics were added to the culture mediums as necessary: for *E. coli*, 20 mg/L of kanamycin (Km) or 100 mg/L of ampicillin; for *P. thermoglucosidasius*, 12.5 mg/L of Km.

### 2.2 Plasmid construction

The primers used in this study were listed in Table S2. For assessing the ligation activity of NHEJ enzymes, the plasmid pZL02-NHEJ was utilized. This plasmid employs pZL02 as its backbone, mirroring the functionality of pZH01, a construct previously developed by our laboratory [20]. For entailed information on pZL02 sequence, refer to Supplementary Note 1. Initially, fragments of NHEJ_Bme genes or NHEJ_Bth genes (Supplementary Note 1) were synthesized by Tsingke Biotechnology Co., Ltd. and amplified using primer pairs Bme-F/Bme-R or Bth-F/Bth-R, respectively. These NHEJ_Bme or NHEJ_Bth fragments, along with plasmid backbone amplified from the pZL02 using primer pair Vector-F/Vector-R, were combined using Gibson assembly to creat the plasmid pZL02-NHEJ_Bme or pZL02-NHEJ_Bth.

To construct the corresponding editing plasmids, gRNAs used to cleavage genome were produced by annealing their corresponding primer pairs. These gRNAs were inserted into the BsaI-digested pZL02-NHEJ plasmid using T4 DNA ligase to generate corresponding editing plasmids, such as pZL02-NHEJ_Bme-gRNA A, containing gRNAs.

### 2.3 Competent cell preparation

Competent cell preparation of *P. thermoglucosidasius* NCIMB 11955 was conducted as described previously [20]. Below is a concise overview of the process. The strain was sub-cultured for three generations on pre-warmed TSA plates at 60 °C. 3–4 single colonies were inoculated into pre-warmed liquid mLB medium and cultivated at 60 °C with shaking for ∼10 h to produce a seed liquid. A portion of the seed solution was transferred into a conical flask containing mLBS medium (adding 0.5 M sorbitol to mLB) and adjusted to an OD_600_ of 0.1. After reaching an OD_600_ level of 1.8 (±0.1), glycine (2 %), dl-alanine (1 %), and Tween 80 (0.0375 %) were added, and the cells were cultured for an additional hour. The flask was then cooled on ice, and the cells were harvested by centrifugation, washed 4 times with ice-cold SMGT medium (0.5 M Sorbitol; 0.5 M Mannitol; 10 % v/v Glycerol; 0.5 M Trehalose), and resuspended in ice-cold SMGT solution. Finally, aliquots were stored at −80 °C.

### 2.4 Phylogenetic tree construction of Ku protein

Initiating our search with the amino acid sequence of the Ku protein from *Bacillus. smithii* ET 138 as a reference [28], we utilized the NCBI database to find over 1,000 sequences with homology. Our next step was to narrow down this list by focusing on sequences that shared more than 45 % identity. To efficiently sift through the homologous sequences for active NHEJ enzyme testing, we applied CD-HIT, a program designed to cluster proteins based on sequence identity, with a cutoff set at 75 %. This clustering allowed us to represent each group by a single sequence, thereby reducing our dataset to 68 unique sequences. We constructed a phylogenetic tree from these selected sequences using MEGA software, employing its neighbor-joining algorithm under default parameters. For the final step of visualizing this tree, we utilized iTOL, an online tool available at http://itol.embl.de.

### 2.5 Genomic long-range deletions with diverses lengths

In the process of editing the genome of *P. thermoglucosidasius* NCIMB 11955, the strain harboring the editing plasmid was initially grown on TSA agar plates supplemented with 12.5 mg/L Km at 52 °C. Subsequently, a single clone from the TSA agar plate underwent PCR verification to ensure the plasmid's integrity, confirming its absence of fragment loss. Following this, the same single clone was cultured in mLB medium supplemented with 50 mM xylose, 10 g/L glucose, and Km at 52 °C with agitation at 250 rpm for 12–24 h. These liquid cultures were then subcultured under the same conditions for 12–24 h and serially diluted before being plated on TSA agar plates containing Km and 50 mM xylose at 52 °C for 12 h. Clones were authenticated through colony PCR and Sanger sequencing. To cure the editing plasmid in strain *P. thermoglucosidasius* NCIMB 11955, a single edited clone, including the editing plasmid, was cultured in mLB medium overnight at 68 °C without Km and then diluted and spread on TAS agar plate. The colonies that grew on TSA plates were randomly picked and screened on TSA plates carrying Km. The colonies that were sensitive to Km were free of editing plasmid.

## 3 Results

### 3.1 Discovery of thermostable NHEJ enzymes

**Fig. 1:**
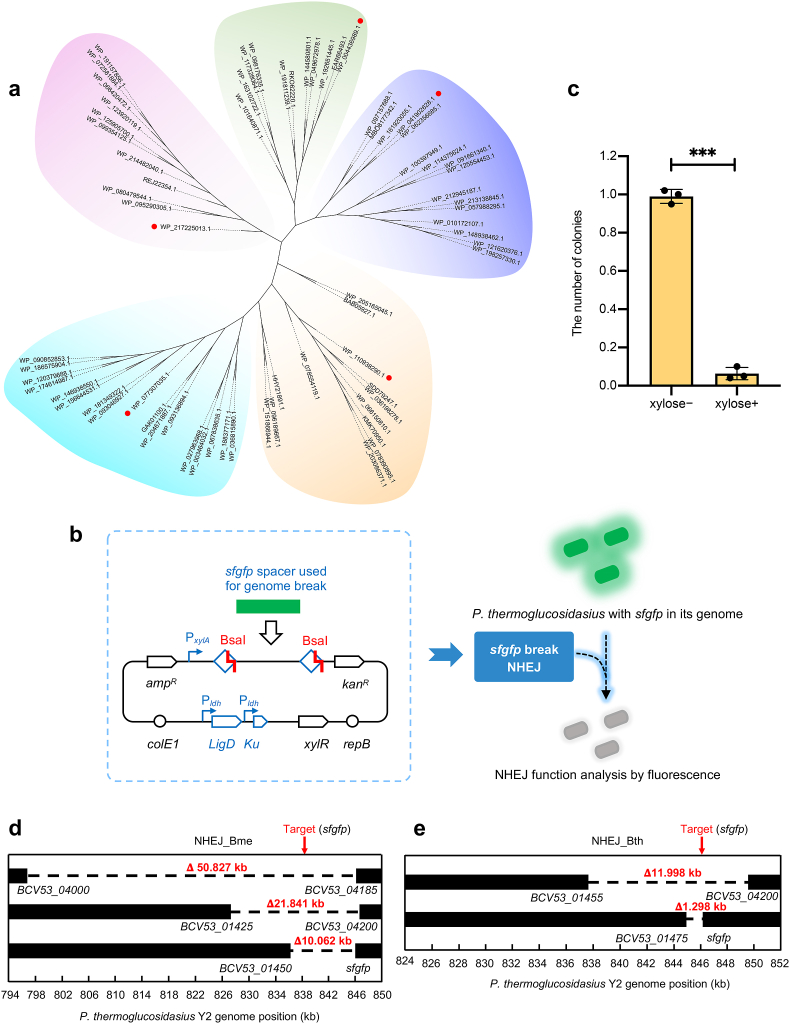
Identification of thermostable NHEJ enzymes. (a) Phylogenetic analysis of Ku proteins was conducted using MAGE software for sequence comparison and tree construction. The neighbor-joining algorithm was employed to build the evolutionary tree. Sequences examined in this study are denoted by red dots. (b) Overview of the assay system used to evaluate NHEJ system activity. (c) Assessing cleavage activity of type I-B CRISPR system activated by xylose. (d) Analysis of deletion size distribution in three independently created *sfgfp* deletion strains conducted via Sanger sequencing. (e) Similar to (d), except with NHEJ_Bme enzymes replaced by NHEJ_Bth. Data are the mean of three biological repeats and are expressed as mean ± SD. Statistical significance is calculated based on two-tailed Student's *t*-test (***P < 0.001).

Thermostable NHEJ enzymes (Ku protein and LigD) are essential to ligate long-range genomic deletion created by type I-B CRISPR system in *P*. *thermoglucosidasius*. In order to obtain thermostable NHEJ enzymes, We survey NHEJ enzymes from thermophilic bacteria reported in published research and find endogenous NHEJ enzymes in thermophilic *Bacillus smithii* growth in 37–65 °C [28]. However, there remains a lack of clarity on the activity of NHEJ enzymes under elevated temperatures. Given that proteins from thermophiles likely possess thermal stability compared with those from mesophiles, we utilized the amino acid sequence of the Ku protein from *B*. *smithii* as a seed sequence for alignment in the NCBI database, noting the essential role of the Ku protein, given that LigC can replace LigD in mycobacteria [26]. This search identified over 1,000 homologous sequences. We then selected homologous sequences with greater than 45 % sequence identity for further analysis. To streamline the active test of NHEJ enzymes, we employed CD-HIT [29] to cluster these proteins with 0.75 threshold for sequence identity, generating 68 clasters. Then we chose the seed sequence from each cluster as a representative, yielding 68 representative sequences (Supplementary Data 1). To further avoid the highly conserved sequences, a phylogenetic tree was constructed from these 68 sequences, which categorized the Ku protein sequences into five evolutionary branches (Fig. 1a). From each branch, one representative Ku protein and its associated LigD enzyme were selected for further testing.

To expedite the assessment of these selected NHEJ enzymes, we devised an assay system that comprises a test plasmid coupled with a Y2 strain [20] that stably harbors the *sfgfp* gene within its genome. In the plasmid, the individual NHEJ enzymes and the xylose-inducible gRNA were designed to simultaneously expresses. The gRNA specifically targeted the *sfgfp* gene for DNA cleavage (Fig. 1b, left panel). When the cleavage activity of the type I-B CRISPR system was activated by xylose and in the absence of NHEJ enzymes, we observed a significant reduction in the number of Y2 colonies (Fig. 1c), indicating that the *sfgfp* gene was processively degraded, yet could not be ligated by the endogenous repair system of *P. thermoglucosidasius*. Conversely, when the plasmid contained a functional NHEJ enzymes, we anticipated the cell death caused by DNA cleavage would be rescued, leading to the appearance of non-fluorescent colonies (Fig. 1b, right panel).

Next, we individually introduced plasmids carrying the representative Ku protein and its associated LigD, along with xylose-inducible crRNA, into the Y2 strain to evaluate the activity of thermostable NHEJ enzyme. After adding xylose to trigger degradation of the *sfgfp* gene within the genome, we only observed a few non-fluorescent colonies on the plates containing the two NHEJ enzymes, namely the Ku proteins WP_004436989.1 (Ku_Bme) and WP_041902628.1 (Ku_Bth) with their cognate LigDs. Subsequent analysis of these non-fluorescent colonies revealed deletions of varying lengths, with the NHEJ_Bme enzymes showing deletions ranging from 10 to 50 kb (Fig. 1d and Fig. S1) and the NHEJ_Bth enzymes showing deletions ranging from 1 to 12 kb (Fig. 1e and Fig. S2). These results indicated that the two NHEJ systems were active.

### 3.2 Improving editing efficiency by adding xylose and glucose

To improve the deletion efficiency, we next checked the whole process for possible causes of low editing efficacy. We first examined the stability of plasmids in fluorescent colonies, finding no loss of fragments related to gRNA or NHEJ enzymes, ruling out plasmid recombination as the cause of the low editing efficiency (Fig. S3a). Despite increasing the promoter strength to potentially boost NHEJ enzyme activity for effective genome ligation, this change did not lead to the appearance of non-fluorescent colonies, suggesting that expression strength of NHEJ enzymes were not the cause of the low editing efficiency (Fig. S3b). Further adjusting the xylose induction concentration also did not improve editing efficiency (Fig. S3c). These results indicated that neither the expression levels of NHEJ enzymes nor the transcription strength of gRNA were limiting factors.

**Fig. 2:**
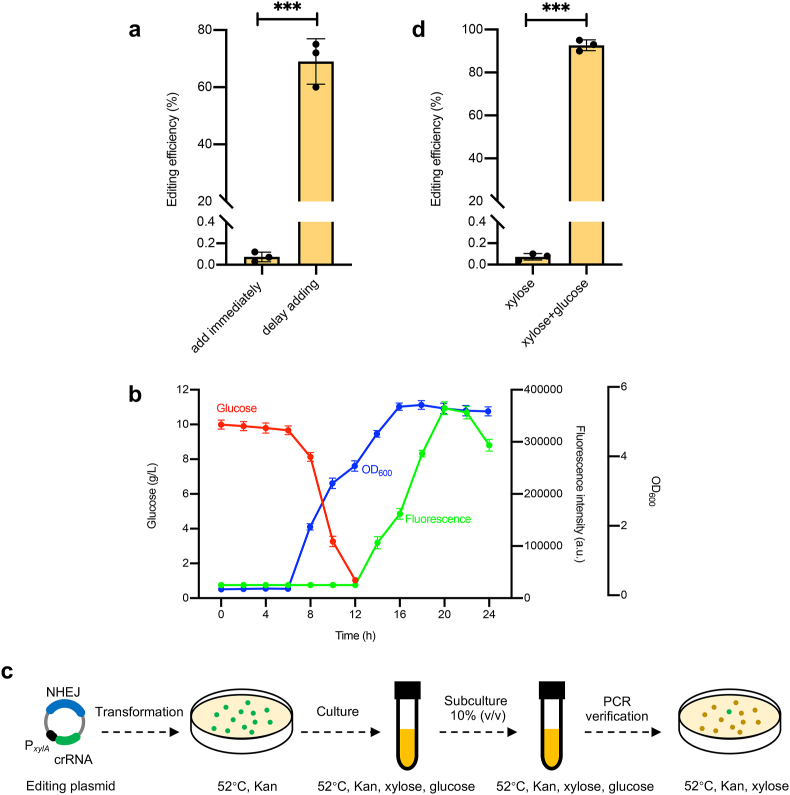
Enhancement of genome editing efficiency with glucose and xylose supplementation. (a) Assessing the editing efficiency by adding xylose into liquid medium either immediately or after a delay. (b) Analysis of the *sfgfp* gene expression driven by a xylose-induced promoter in a liquid medium supplemented with both glucose and xylose. (c) The workflow of genome editing. (d) Comparison of editing efficiencies in strains cultivated in media supplemented with either xylose alone or a combination of xylose and glucose. Data are the mean of three biological repeats and are expressed as mean ± SD. Statistical significance is calculated based on two-tailed Student's *t*-test (***P < 0.001).

Given that the expression strength of NHEJ enzymes and gRNA was not the bottleneck, we hypothesized that the premature activity of gRNA, combined with inadequate NHEJ expression, contributed to the low editing efficiency. To test this hypothesis, we decided to delay the addition of xylose to the culture medium to allow for adequate NHEJ enzyme expression. This adjustment led to a noticeable improvement in editing efficiency (Fig. 2a).

To streamline the operation process, we tried to delay gRNA expression by simultaneously adding glucose and xylose to the medium during the inoculation, leveraging the temporal regulation of glucose catabolite repression [30,31]. To assess if the expression of the xylose-inducible promoter could be postponed by co-addition, a plasmid expressing the *sfgfp* gene under a xylose-inducible promoter was introduced into wild-type strains. We found that *sfgfp* gene expression was initiated only after the complete consumption of the glucose (Fig. 2b). This result demonstrated that the concurrent addition of glucose and xylose effectively delayed the timing of gRNA-mediated genome cleavage.

So we inoculated a single colony from a plate into liquid medium containing both sugars, then subcultured them once under the same conditions, and finally transferred them to a solid medium. We observed that the majority of the resulting colonies were non-fluorescing (Fig. 2c). The editing efficiency reached 92.6 % when using both sugars, in pronounced contrast to 0.07 % with xylose alone (Fig. 2d). This significant advancement demonstrated the effectiveness and simplicity of our autoregulated xylose induction strategy in improving the efficiency of gene editing.

### 3.3 GC content of gRNAs is negatively correlated with editing efficiency of long-range genomic deletions

**Fig. 3:**
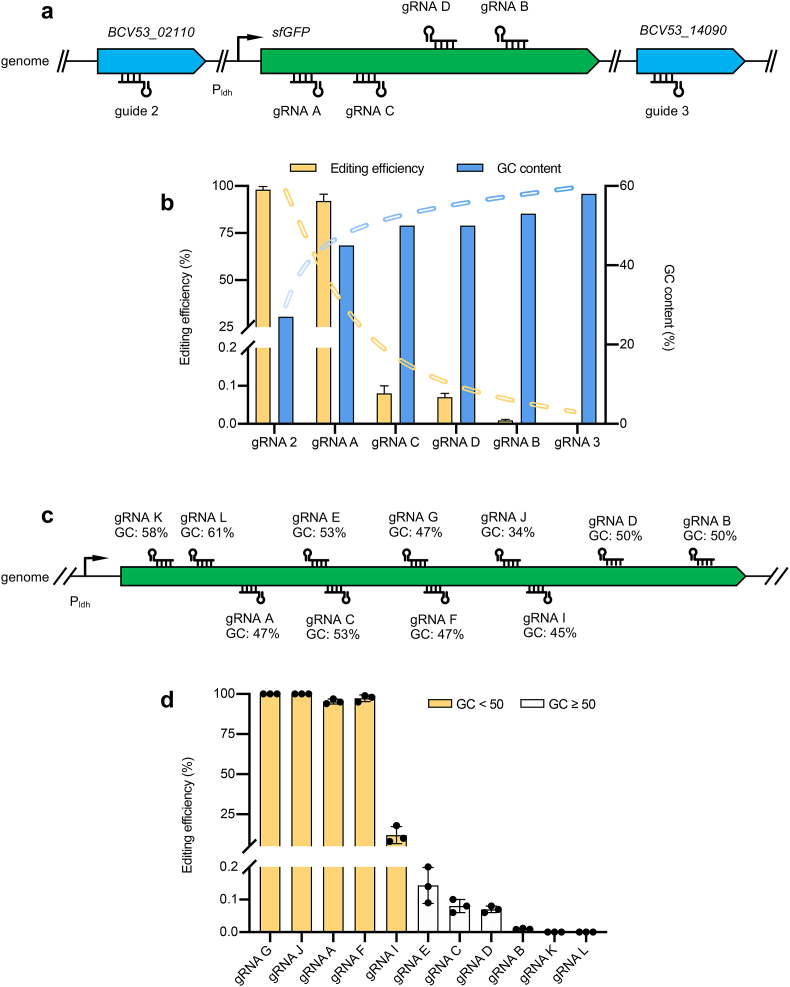
Evaluation of genome editing efficiencies using various gRNAs. (a) Locations of gRNAs across multiple genes. (b) Negative correlation between gRNAs editing efficiency and their GC contents. (c) Selection of gRNAs from *sfgfp* gene based on varying GC contents. (d) Comparison of editing efficiency for gRNAs with diverse GC contents. Data are the mean of three biological repeats and are expressed as mean ± SD.

Given the high editing efficiency achieved with gRNA A targeting *sfgfp* gene by adding both sugars to the medium (Fig. 2d), we expanded our investigation to assess the generalizability of editing efficiency across additional genomic targets. We selected three more gRNAs targeting the *sfgfp* gene (namely, gRNA B, gRNA C, and gRNA D) and two targeting other genes for further testing (Fig. 3a). Contrary to our expectations, we discovered a distinct preference in editing efficiency towards certain targets. Specifically, gRNA 2 exhibited the highest editing efficiency of 98 %, whereas the gRNA 3 showed no editing efficiency at all (Fig. 3b). To understand the underlying patten of target preference, we calculated the GC content of these gRNAs. This analysis revealed a trend where gRNAs with higher editing efficiencies tend to have lower GC contents (Fig. 3b).

To further confirm the trend that gRNA editing efficiency inversely correlated with their GC content, we selected additional gRNAs with varying GC contents from the *sfgfp* gene (Fig. 3c). We found that gRNAs with less than 50 % GC content showed higher editing efficiencies, all exceeding 95 %, except for gRNA I, which had an editing efficiency of 12 %. By contrast, gRNAs with GC content greater than 50 % exhibited extremely low editing efficiency, all below 0.2 % (Fig. 3d). These results indicate that the editing efficiency of gRNAs is indeed target specific, favoring gRNAs with lower GC content for higher efficiencies (Fig. 3d).

To investigate the observation that gRNAs with lower GC content exhibited higher editing efficiencies, we proposed that these gRNAs might have lower melting temperatures (Tm), which could facilitate the detachment of the gRNA-led cascade from its target DNA following cleavage. Such detachment would potentially allow easier access for the NHEJ enzymes to the cleaved DNA ends. To explore this hypothesis, we attempted to lower the Tm values of gRNAs with high GC content. Our previous studies indicated that shortening the spacer length to ≥27 bp did not affect the cleavage function of the type I-B system [20]. Therefore, we shortened the spacer lengths of gRNA C, gRNA K, and gRNA L with GC contents of 53 %, 58 %, and 61 %, respectively, to 27 bp to reduce their Tm values (Fig. S4a), but this shortening did not improve their editing efficiencies (Fig. S4b). This outcome suggested that while the hypothesis regarding Tm values and gRNA detachment post-cleavage was plausible, the strategy of shortening spacer lengths to enhance editing efficiency for high GC content gRNAs did not yield the anticipated results.

### 3.4 Designing genome-wide gRNAs selection manual

**Fig. 4:**
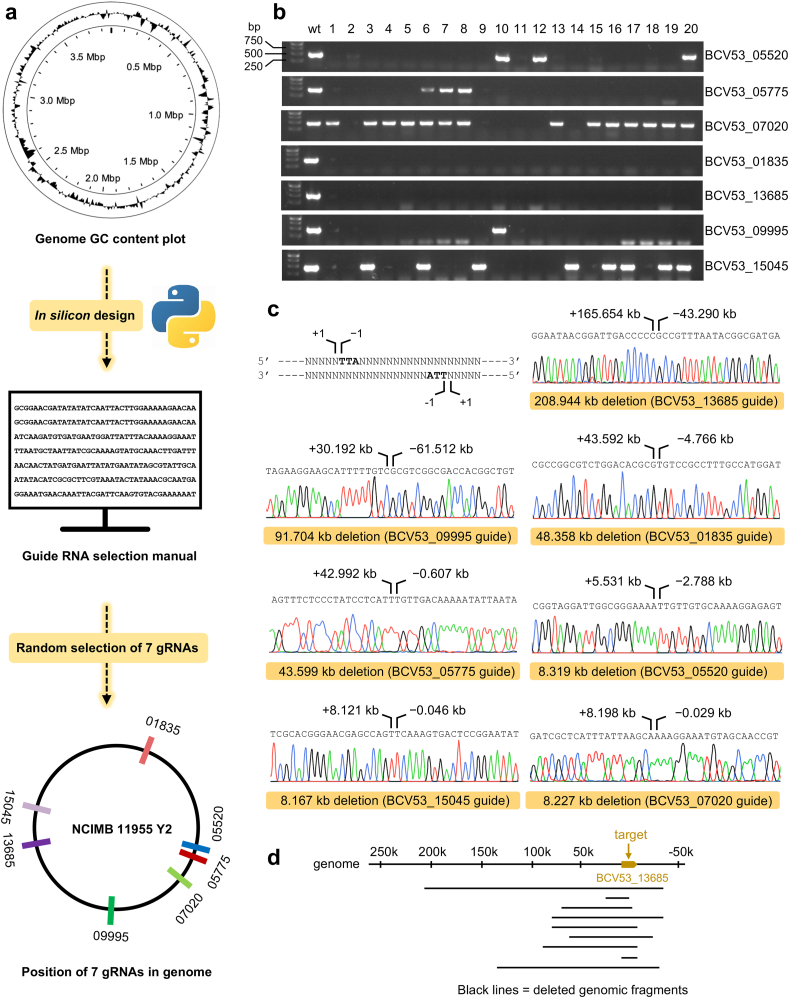
Assessing editing efficiency across the whole genome. (a) Development of a gRNA selection manual for each gene within the genome, followed by testing through the random sampling of several gRNAs. (b) Evaluation of the genome editing efficacy for each of the seven chosen gRNAs. The Y2 parental strain is used as a control (wt). (c) Determination of the maximum deletion lengths achieved by each of the seven chosen gRNAs through sanger sequencing. The bold TTA sequence indicated the PAM sequence. (d) A gRNA targeting BCV53_13685 gene generating large genomic deletions with varying lengths.

Although gRNAs with low GC contents exhibited high editing efficiencies, selecting these gRNAs from genes within the *P. thermoglucosidasius* genome is time-consuming and labor-intensive due to the variable GC content distribution across the genome (Fig. 4a). To streamline the selection process, a Python script was utilized to select the three gRNAs with the lowest GC content for each gene (Supplementary Note 2). This approach generated a comprehensive gRNA selection manual for the entire genome (Supplementary Data 2). To assess the effectiveness of our designed manual, seven gRNAs targeting respective genes were randomly selected from the manual to test their editing efficiency (Fig. 4a and Table S3). Gene editing was carried out following the workflow illustrated in Fig. 2c, and 20 colonies were validated for each selected gRNA. We observed that all seven gRNAs can accomplishing gene editing, with the highest editing efficiency of one gRNA reaching 100 % (Fig. 4b). This result strongly indicates the high efficiency of our designed gRNA selection manual.

Given the existing knowledge gap regarding essential and non-essential genes for *P. thermoglucosidasius*, the maximum deletion length achieved by each of the seven gRNAs was also determined. This effort aimed to compile a preliminary list of non-essential genes. The sequencing data indicated that the gRNA targeting BCV53_13685 achieved the longest deletion of 208.944 kb, and all seven gRNAs achieved more than 8 kb deletion (Fig. 4c). To calculate all non-essential genes in this study, we also consider the deletion length obtained by gRNA A, which achieved a maximum deletion length of 50.827 kb (Fig. 1d). Collectively, the cumulative deletion length of these eight gRNAs was 468.145 kb, accounting for 12 % of the total genomic length. The initial compilation of non-essential genes is provided (Supplementary Data 3).

Considering that genomic deletion lengths were a spectrum by type I-B system in our research (Fig. 1d and e) and other research, we further examined different deletion lengths induced by a single gRNA. Selecting the gRNA targeting BCV53_13685 gene as a representative, the genomic deletion lengths of several randomly selecting strain were analyzed by sanger sequencing (Fig. 4d and Fig. S5). This analysis confirmed that a long-range genomic deletions could be achieved by a single gRNA from our selection manual, highlighting the exceptional utility of manual in facilitating effective genome editing.

## 4 Discussion

Existing tools for precise large genomic deletions utilizing the type I CRISPR system have been developed by employing the nCas3 nickase in conjunction with the host's homology-directed repair system [32]. However, long-range genomic deletions of variable lengths utilizing the type I CRISPR system have only been implemented in human cells or prokaryotic cells possessing endogenous NHEJ or AJ repair system. The absence of repair systems, especially for NHEJ enzymes, have limited the ability of the endogenous type I CRISPR-Cas system to generate a variety of extensive range genomic deletions in bacteria. This limitation is particularly remarkable given that the type I system is the most prevalent CRISPR-Cas system [33]. In our study, two active NHEJ enzymes were identified and characterized from thermophilic bacteria, presenting the first demonstration of NHEJ enzymes functioning at elevated temperatures (Fig. 1d and e). Considering the widespread utilization of endogenous or heterogenous type I and II systems in thermophiles [16,20,[34], [35], [36]], our discoveries provide essential components for developing extensive range genomic deletion tools or donor DNA-free gene knockout tools in other biotechnological potential thermophilic strains [37].

The synergy between CRISPR-Cas systems and NHEJ repair system is critical for achieving highly-efficient genetic engineering. Research by Yan et al. found that inhibiting RecA-dependent homologous recombination could enhance the ligation efficiency of NHEJ, thus increasing Cas9-mediated genome editing efficiency in *Mycobacterium tuberculosis* [38]. Similarly, David Bikard and colleagues demonstrated a competitive interaction between the Csn2 protein of endogenous type II CRISPR system in *Staphylococcus aureus* and NHEJ enzymes over binding to cleaved DNA, which decreased gene editing efficiency [39]. In eukaryotic cells, modifications to sgRNA, such as mutations or truncations, were found to facilitate Cas9 dissociation, providing substrates for NHEJ and enhancing its efficiency [40]. However, these studies all focused on the Type II systems, leaving the interaction between type I CRISPR systems and NHEJ enzymes largely unexplored. Our research fills this gap by demonstrating a distinct affinity of the NHEJ repair pathway for rejoining DNA fragments generated by genomic cleavage mediated by low-GC-content gRNAs within the Type I-B CRISPR system (Fig. 3).

Echoing the insights of modifications to sgRNA in eukaryotic cells, we hypothesized that gRNAs with lower GC content could possess lower Tm values, potentially facilitating the release of the gRNA-guided complex from its target DNA post-cleavage. Such a release could theoretically provide NHEJ enzymes with better access to the cleaved DNA ends. Despite this, our attempts to improve editing efficiency by truncating gRNAs within the Type I CRISPR system did not yield positive results (Figs. S4a and b). This outcome diverges from the expectations set by observations in Type II systems and suggests that the more intricate effector proteins in the Type I system may contribute to more complex interactions. For example, the type I-B CRISPR system in *P. thermoglucosidasius* might involve effector complexes that act as single-turnover enzymes, similar to Cas9 [41], possibly preventing NHEJ enzymes from effectively ligating the cleaved genome. Considering the diminished editing efficiency encountered with high-GC-content targets in type I systems, investigating strategies such as the knockout of Cas4 (homologous protein of Csn2) and RecA—strategies proven to enhance linkage efficiency in Type II systems—could be a promising direction for improving editing outcomes with high-GC-content DNA targets.

While the Type I-B system, in combination with NHEJ enzymes, facilitates long-range genomic deletions of varying lengths in *P. thermoglucosidasius*, the broader capabilities of type I CRISPR systems have yet to be fully leveraged. This includes the development of long-range CRISPR screens that can efficiently construct comprehensive deletion libraries with the use of minimal gRNAs. This approach, simpler and more cost-effective compared to the elaborate and expensive CRISPR interference (CRISPRi) libraries, enables the detailed exploration of long-range genotype-phenotype correlations. Considering the proven effectiveness of our tools at the whole-genome level and the development of a comprehensive gRNA selection manual (Fig. 4), future research could pivot towards establishing an extensive deletion library that encompasses the whole genome. Such a library would have broad utility in various fields, including functional genomics, the analysis of gene interaction networks, the streamlining of genomes, and the optimization of cell factories, showcasing the significant application potential of this technology in scientific research and biotechnological innovation.

## CRediT authorship contribution statement

**Zhiheng Yang:** Conceptualization, Data curation, Investigation, Methodology, Software, Formal analysis, Visualization, Writing – original draft, Writing – review & editing. **Bixiao Li:** Formal analysis, Methodology, Validation. **Ruihong Bu:** Methodology, Validation. **Zhengduo Wang:** Data curation, Software. **Zhenguo Xin:** Data curation, Software. **Zilong Li:** Resources, Supervision. **Lixin Zhang:** Conceptualization, Supervision. **Weishan Wang:** Conceptualization, Resources, Supervision, Funding acquisition, Writing – original draft, Writing – review & editing.

## Declaration of competing interest

We hereby affirm that there are no commercial or associative interests that could be construed as a conflict of interest with the submitted work.
