# Supplementary material for: A highly efficient method for genomic deletion across diverse lengths in thermophilic Parageobacillus thermoglucosidasius
Source: Synth Syst Biotechnol. 2024 May 17;9(4):658–66. doi: 10.1016/j.synbio.2024.05.009 (PMC11137367; doi:10.1016/j.synbio.2024.05.009)
Supplement: Multimedia component 1 [file mmc1.pdf]

Supplementary information

A highly efficient method for genomic deletion across diverse lengths in thermophilic *Parageobacillus thermoglucosidasius*

This includes detailed supporting results, materials utilized, and DNA sequences. It encompasses Fig. S1–S5, Tables S1–S3, and Supplementary Notes 1–Note 2. Supplementary Data: This included Ku protein sequences used to conduct phylogenetic tree analysis, the whole-genome gRNA selection manual, and the non-essential genes in *P. thermoglucosidasius*. It encompasses Supplementary Data 1–Data 3, which are provided in Excel (xlsx) format.

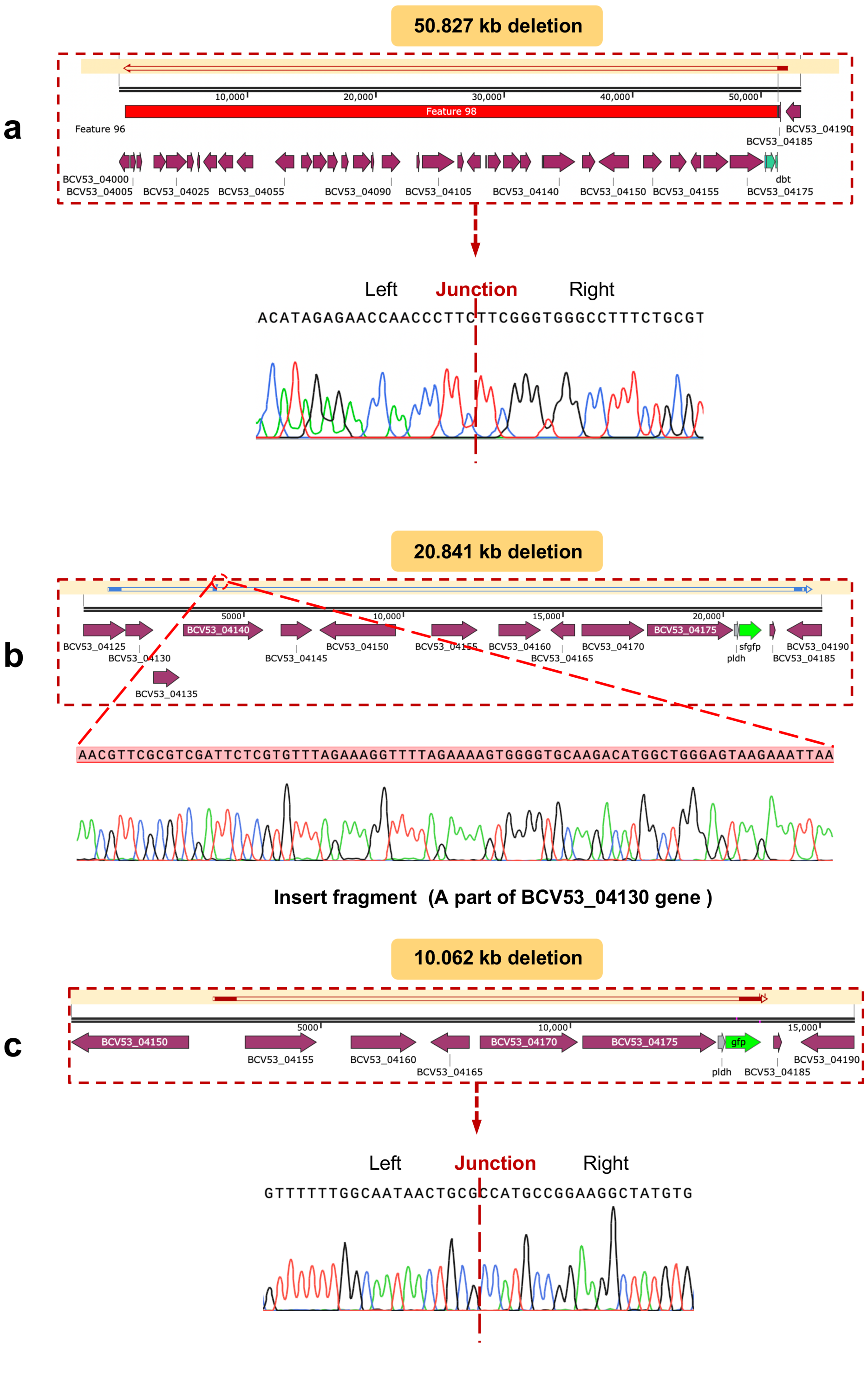

Fig. S1. Sequencing of genomic deletion length in three non-fluorescent colonies using Type I CRISPR paired with NHEJ_Bme enzymes. Panels a, b, and c correspond to three different colonies, with deletions measuring 50.827 kb, 20.841 kb, and 10.062 kb, respectively, as determined by sanger sequencing.

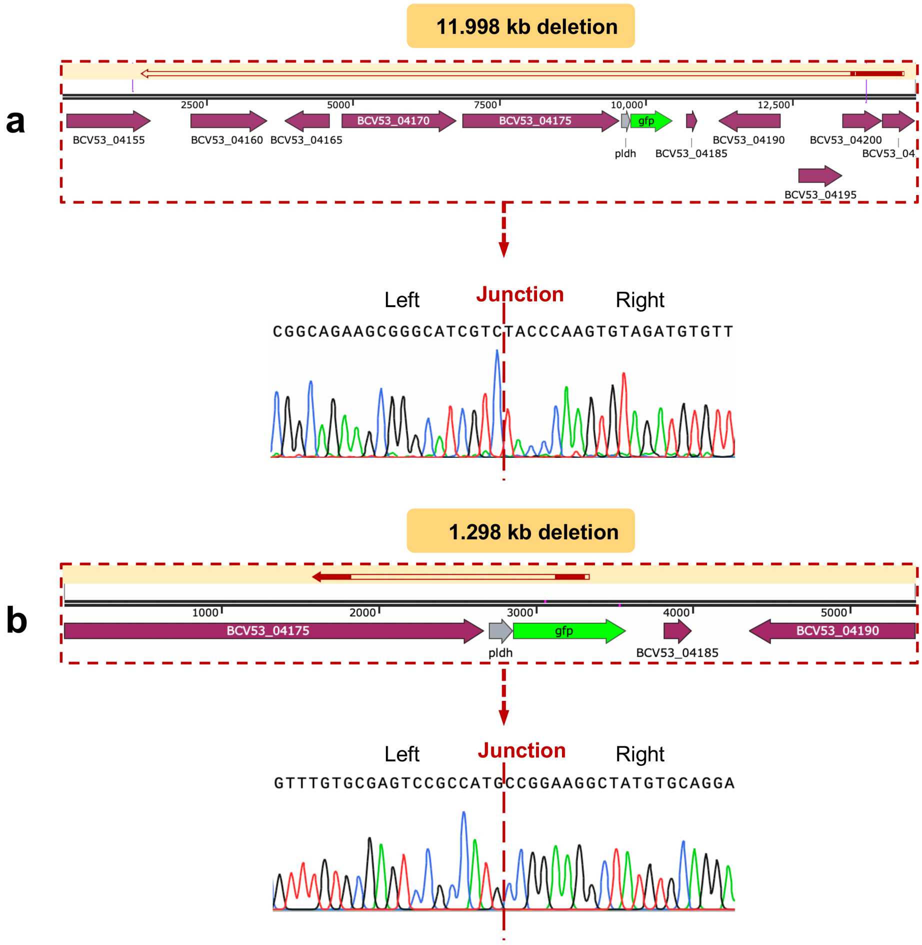

Fig. S2. Sequencing of genomic deletion length in two non-fluorescent colonies using Type I CRISPR paired with NHEJ_Bth enzymes. Panels a and b correspond to two different colonies, with deletions measuring 11.998 kb and 1.298 kb, respectively, as determined by sanger sequencing.

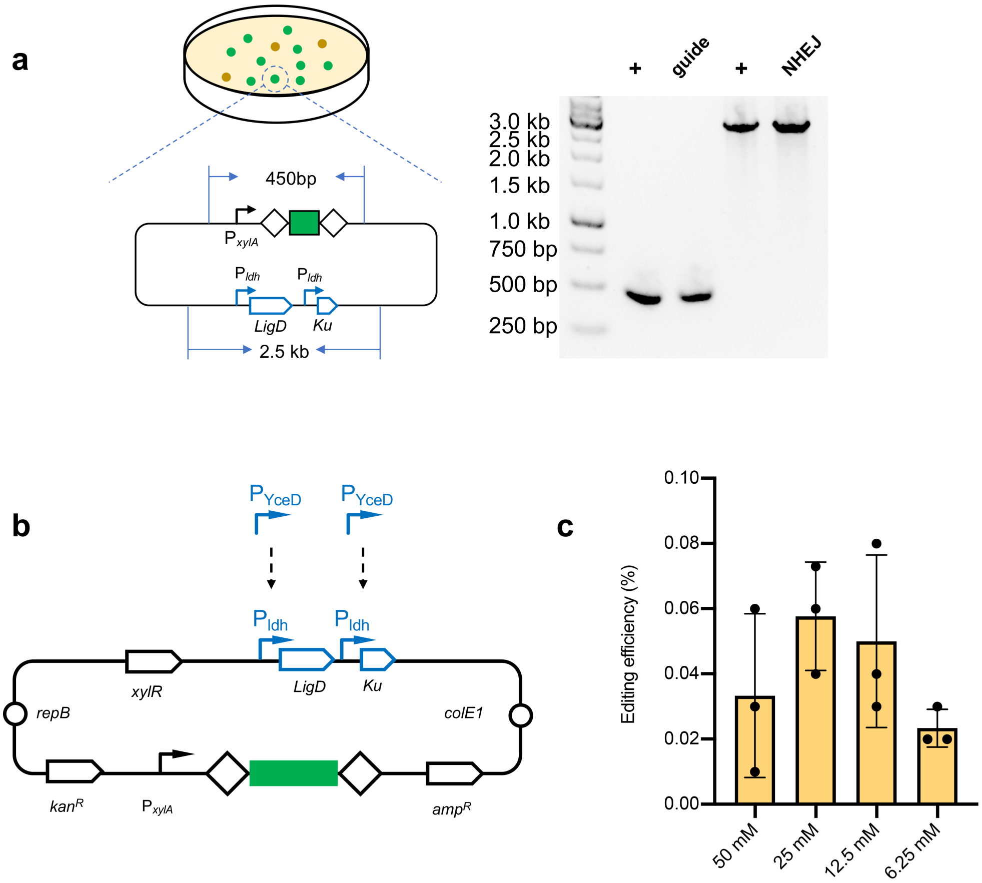

Fig. S3. Investigating the cause of low editing efficiency. (a) Evaluation of plasmid stability by PCR analysis. Left panel: Schematic of editing plasmid containing gRNA and NHEJ enzymes. Right panel: gRNA length, 450 bp; NHEJ enzymes length, 2.5 kb. (b) Replacing P_{ldh} promoter with stronger P_{Yced} promoter. (c) Assessment of xylose concentration impact on editing efficiency.

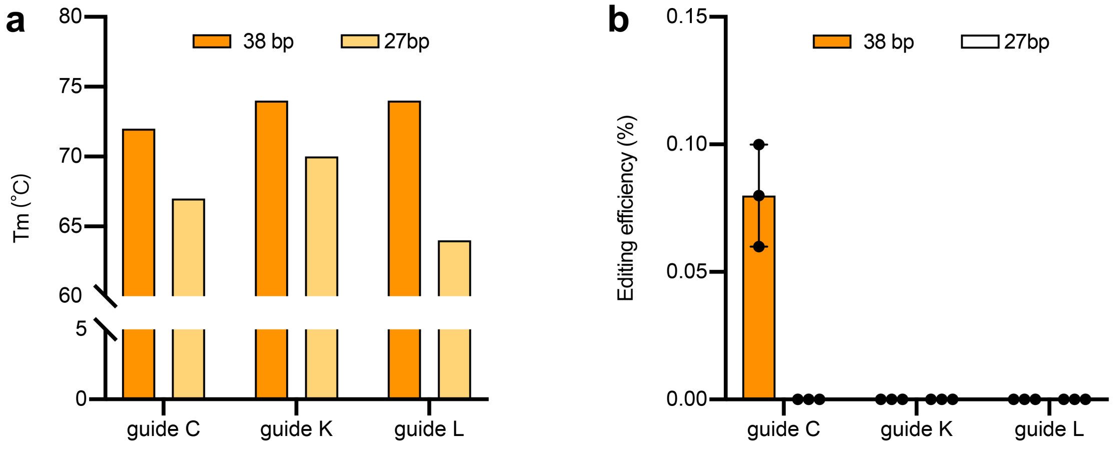

Fig. S4. The impact of gRNA Tm value on editing efficiency. (a) Reduction in Tm value of gRNA due to spacer truncation. (b) Comparison of gRNA editing efficiencies before and after spacer truncation.

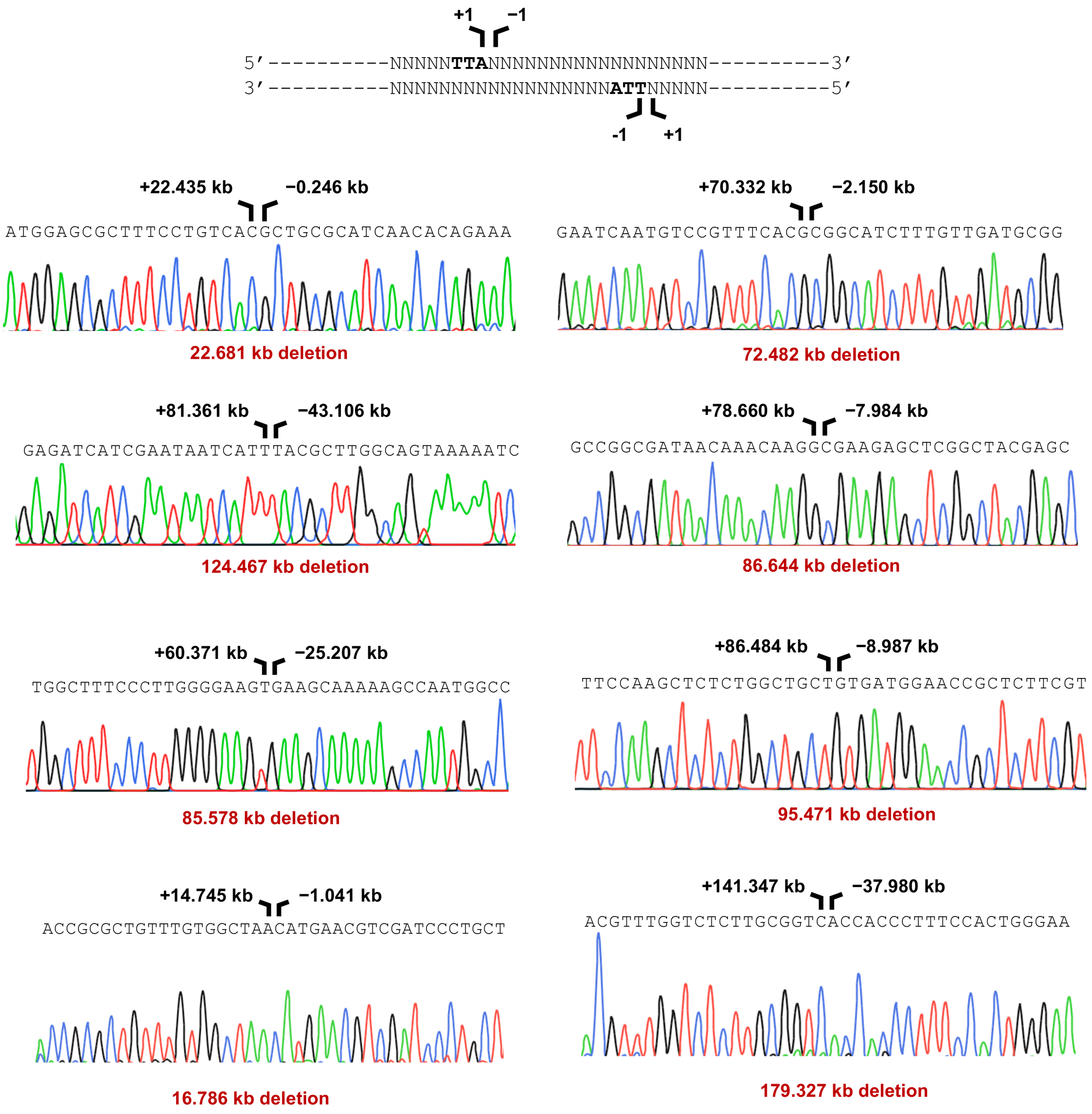

Fig. S5. Sequencing genomic deletion lengths with varying length by the gRNA targeting BV53_13685 gene.

Table S1 Strains used in this study

Strains	Description	Source
E. coli JM109	General cloning host for plasmid manipulation	Lab stock
Parageobacillus thermoglucosidasius NCIMB 11955	Wild type	Lab stock
P. thermoglucosidasius Y2	Deleting all type III-B Cas proteins and introducing *sfgfp* reporter gene into genome	Lab stock

Table S2 Primers used in this study

Name	Sequence (5’-3’)
For pZL02-NHEJ_Bme or pZL02-NHEJ_Bth construction	
vector-F	tggcgtaatcatggtcatagc
vector-R	ctatgaaatgacttctaactccgaagg
Bme-F	gagttagaagtcatttcataggtaaaacgacggccagt
Bme-R	ctatgaccatgattacgccacttaagttacgcttctctctttg
Bth-F	gagttagaagtcatttcataggtaaaacgacggccagt
Bth-R	ctatgaccatgattacgccactaagcttttttcttggcacggg
For editing plasmid, i.e., inserting corresponding gRNA into pZL02-NHEJ	
guide A-F	gaaactggtgttcagtgctttgctcgttatccggaccatatgag
guide A-R	caaactcatatggtccggataacgagcaaagcactgaacaccag
guide B-F	gaaaccgaactccagcagaaccatatgatcgcgtttctcgttcg
guide B-R	caaacgaacgagaaacgcgatcatatggttctgctggagttcgg
guide C-F	gaaacaggatgacggcacgtacaaaacgcgtgcggaagtgaaag
guide C-R	caaactttcacttccgcacgcgttttgtacgtgccgtcatcctg
guide D-F	gaaacgacagaacgctttgcgtgctcagatagtgattgtctggg
guide D-R	caaacccagacaatcactatctgagcacgcaaagcgttctgtcg
guide E-F	gaaacaaggaaatcgtgcgttcctgcacatagccttccggcatg
guide E-R	caaacatgccggaaggctatgtgcaggaacgcacgatttccttg
guide F-F	gaaacaagaagacggcaatatcctgggccataagctggaatacg
guide F-R	caaacgtattccagcttatggcccaggatattgccgtcttcttg
guide G-F	gaaacaagtcaatgcctttcagctcaatgcggtttaccagggtg
guide G-R	caaacaccctggtaaaccgcattgagctgaaaggcattgacttg
guide I-F	gaaacaagcgaattttaaaattcgccacaacgtggaggatggcg
guide I-R	caaacgccatcctccacgttgtggcgaattttaaaattcgcttg
guide J-F	gaaacatgccattttttttgtttatcggcggtgatgtaaacattg
guide J-R	caaacaatgtttacatcaccgccgataaacaaaaaaaatggcatg
guide K-F	gaaacgttgcgtcaccttcaccctcgccacgcacggaaaacttg

guide K-R	caaacaagttttccgtgcgtggcgagggtgaaggtgacgcaacg
guide L-F	gaaacccattagttgcgtcaccttcaccctcgccacgcacggag
guide L-R	caaactccgtgcgtggcgagggtgaaggtgacgcaactaatggg
guide 2-F	gaaacatttgaaacatattcgagagaaaaatgaagaactttggg
guide 2-R	caaacccaaagttcttcatttttctctcgaatatgtttcaaatg
guide 3-F	gaaacatctgcaaaatggcgatgccgtccgctttttcgcgcgcg
guide 3-R	caaacgcgcgcgaaaaagcggacggcatcgccattttgcagatg
guide 01835-F	gaaacttaatgctaattatcgcaaaagtatgcaaacttgatttg
guide 01835-R	caaacaaatcaagtttgcatacttttgcgataattagcattaag
guide 05520-F	gaaacgcggaacgatatatatcaattacttggaaaaagaacaag
guide 05520-R	caaacttgttctttttccaagtaattgatatatatcgttccgcg
guide 05775-F	gaaacctttatcgtattgcatttttcttcataaaaacgatgtg
guide 05775-R	caaacacatcgtttttatgaagaaaaaatgcaatacgataaagg
guide 07020-F	gaaacatcaagatgtgatgaatggattatttacaaaaggaaatg
guide 07020-R	caaacatttccttttgtaaataatccattcatcacatcttgatg
guide 09995-F	gaaacaacaagaaattaatagcttccataaaaatgatgaattag
guide 09995-R	caaactaattcatcatttttatggaagctattaatttcttgttg
guide 13685-F	gaaacatatacatcgcgcttcgtaaatactataaacgcaatgag
guide 13685-R	caaactcattgcgtttatagtatttacgaagcgcgatgtatatg
guide 15045-F	gaaacggaaatgaacaaattacgattcaagtgtacgaaaaaatg
guide 15045-R	caaacatttttcgtacacttgaatcgtaatttgttcatttccg

Table S3 Selected seven spacer sequences of gRNAs from the manual

spacer sequence (38 bp)	Targeted gene name
ttaatgctaattatcgcaaaagtatgcaaacttgattt	BCV53_01835
gcggaacgatatatatcaattacttggaaaaagaacaa	BCV53_05520
ctttatcgtattgcattttttcttcataaaaacgatgt	BCV53_05775
atcaagatgtgatgaatggattatttacaaaaggaaat	BCV53_07020
aacaagaaattaatagcttccataaaaatgatgaatta	BCV53_09995
atatacatcgcgcttcgtaaatactataaacgcaatga	BCV53_13685
ggaaatgaacaaattacgattcaagtgtacgaaaaaat	BCV53_15045

Supplementary Note 1

The specific sequence of pZL02 plasmid

ccacagcttgtctgtaagcggatgccgggagcagacaagcccgtcagggcgcgtcagcgggtgttggcgggtgtcggg
gctggcttaactatgcggcatcagagcagattgtactgagagtgcaccatatatgcggtgtgaaataccgcacagatgcgta
aggagaaaataccgcatcaggcgtaaccaacatgattaacaattattagaggtcatcgttcaaaatggtatgcgttttgacac
atccactatatatccgtgtcgttctgtccactcctgaatcccattccagaaattctctagcgattccagaagtttctcagagtcgg
aaagttgaccagacattacgaactggcacagatggtcataacctgaaggaagatctgattgcttaactgcttcagttaagacc
gaagcgctcgtcgtataacagatgcgatgatgcagaccaatcaacatggcacctgccattgctacctgtacagtcaaggat
ggtagaaatgttgtcggtccttgcacacgaatattacgccatttgcctgcatattcaaacagctcttctacgataagggcacaa
atcgcatcgtggaacttttgggcttctaccgatttagcagtttgatacactttctctaagtatccacctgaatcataaatcggcaa
aatagagaaaaattgaccatgtgtaagcggccaatctgattccacctgagatgcataatctagtagaatctcttcgctataaaa
attcacttccaccttccactcaccggttgtccattcatggctgaactctgcttcctctgttgacatgacacacatcatctcaatatc
cgaatagggcccatcagtctgacgaccaagagagccataaacaccaatagccttaacatcatccccatatttatccaatattc
gttccttaatttcatgaacaatcttcattctttcttctctagtcattattattggtccattcactattctcattcccttttcagataatttta
gatttgcttttctaaataagaatatttggagagcaccgttcttattcagctattaataactcgtcttcctaagcatccttcaatcctttt
aataacaattatagcatctaatcttcaacaaactggcccgtttgttgaactactctttaataaaataatttttccgttcccaattcca
cattgcaataatagaaaatccatcttcatcggctttttcgtcatcatctgtatgaatcaaatcgccttcttctgtgtcatcaaggttt
aattttttatgtatttcttttaacaaaccaccataggagattaaccttttacggtgtaaaccttcctccaaatcagacaaacgtttca
aattcttttcttcatcatcggtcataaaatccgtatcctttacaggatattttgcagtttcgtcaattgccgattgtatatccgatttat
atttatttttcggtcgaatcatttgaacttttacatttggatcatagtctaatttcattgcctttttccaaaattgaatccattgtttttgat
tcacgtagttttctgtattcttaaaataagttggttccacacataccaatacatgcatgtgctgattataagaattatctttattattta
ttgtcacttccgttgcacgcataaaaccaacaagatttttattaattttttttatattgcatcattcggcgaaatccttgagccatatct
gacaaactcttatttaattcttcgccatcataaacatttttaactgttaatgtgagaaacaaccaacgaactgttggcttttgtttaa
taacttcagcaacaaccttttgtgactgaatgccatgtttcattgctctcctccagttgcacattggacaaagcctggatttacaa
aaccacactcgatacaactttctttcgcctgtttcacgattttgtttatactctaatatttcagcacaatcttttactctttcagcctttt
aaattcaagaatatgcagaagttcaaagtaatcaacattagcgatttttcttttctctccatggcctcacttttccacttttttgtcttgt
ccactaaaacccttgatttttcatctgaataaatgctactattaggacacataatattaaaagaaacccccatctatttagttatttg
tttagtcacttataactttaacagatggggtttttctgtgcaaccaattttaagggttttcaatactttaaaacacatacataccaac
acttcaacgcacctttcagcaactaaaataaaaatgacgttatttctatatgtatcaagataagaaagaacaagttcaaaaccat
caaaaaaagacaccttttcaggtgcttttttttattttataaactcattccctgatctcgacttcgttcttttttacctctcggttatgag
ttagttcaaattcgttctttttaggttctaaatcgtgtttttcttggaattgtgctgttttatcctttaccttgtctacaaaccccttaaaa
acgtttttaaaggcttttaagccgtctgtacgttccttaaggcaagttcctttcgccgcaaaaatgtaaaagaaagggattcatc
tgtttttttattgacagaaaaactatgttagtttagtatataaactaatgtaatgaaacagaaacagtgattcatttttatgtttgcaca
tcatcggaggcagaaaagacggggcaacaccgattgaatggggtgcggaatccgcgttggctgtgggcgcttggcggat
cataggaaaaagtaaacgtgtgtttgcatatgtcatgtcccagagataaactttttatacagccgctcagaggttgatccgcct
gtttaggcggcatactaaaaaggttgcagaaggaatgagtgaatatggtgcgaacaggaaatataggactcgtcaaaaaaa
tcaataaacagatcgtattaaagctgattcgcgaaaaaaataacatttcgcgggcggagatcgccaaaatcaccggattaaa
caaggcgacggtgtctgcgttagtcgatgaattaatttccgaacattttgtcagtgaaagcggaattggcgtttccaccggcg
gacgccggccgcttatgcttcgttttaacgaagcggcaggctcattaatcggcatagagttgggggttaactacatttatgcc
gtattaacagacttaaacgccgagatcatttggcaaaagatggtccacttccgcgccaatgaaacgcaggaaacaatcatg
gaaaaaatgatcgcgatgattcatgaagccatccgccatgcgccagctactccgtatggaatcatgggcatcggtattggc
gtgccggggattgtgaacacagaggaaggagttgtcatatttgccccaaaccttcattgggatcatgttgcccttgcttccatt
ctgcaaaaacaatggccggattatccgattattattgaaaacgaagcaaaactggcggcgcttggcgagaaatggtttggc

gcgggcaaagagtttgccaattttgtatatgttagcgccggaaccgggattggcgccgggatcatcattcacaaccagctgt
accgcggtaccgatggcattgccggcgaaatcggccatcatgtcattgacattcatggcgttcgctgcagctgcggcaata
acggatgctgggagatgtacgcgtcagaaaaatatattcaacgccggctcgagcaggaaaatcatcattccatgctagaag
acttttccgtagaaaaagtgcgtgcattggctgagcgcggcgacaagcaaatggcgcaaatattggcggaggtcggccgc
tacttggggattggcattttacatatcatttatgcctataacccggaagccgtcattgtcggcaatacgattggcacggcaggc
aagtgggtgctggagccggcacgggacgaagtcgcaaaaaggatattggtgaaaaatgggggcggcccgttcattattc
catcgcagctatcggaaaaaagctgtgcaattggcgccgtggcttccgtattagaaaaagtagtgatgccttcggagttaga
agtcatttcatagcagtctaatcaggtgaaaaaaaaaccccgcccctgacagggcggggtttttttttctagagtcgactcact
tgtacagctcgtccatgccgccggtggagtggcggccctcggcgcgttcgtactgttccacgatggtgtagtcctcgttgtg
ggaggtgatgtccaacttgatgttgacgttgtaggcgccgggcagctgcacgggcttcttggccttgtaggtggtcttgacct
cagcgtcgtagtggccgccgtccttcagcttcagcctctgcttgatctcgcccttcagggcgccgtcctcggggtacatccg
ctcggaggaggcctcccagcccatggtcttcttctgcattacggggccgtcggaggggaagttggtgccgcgcagcttca
ccttgtagatgaactcgccgtcctgcagggaggagtcctgggtcacggtcaccacgccgccgtcctcgaagttcatcacgc
gctcccacttgaagccctcggggaaggacagcttcaagtagtcggggatgtcggcggggtgcttcacgtaggccttggag
ccgtacatgaactgaggggacaggatgtcccaggcgaagggcaggggggccaccttggtcaccttcagcttggcggtct
gggtgccctcgtaggggcggccctcgccctcgccctcgatctcgaactcgtggccgttcacggagccctccatgtgcacc
ttgaagcgcatgaactccttgatgatggccatgttatcctcctcgcccttgctcaccatggatcccatttttttcctcctttttcg
atgctttttcttttataagcttgggtagaatattaagttaattatatcacatgctaaattcagtgtcaaggagggcgcgcctggc
gtaatcatggtcatagctgtttcctgtgtgaaattgttatccgctcacaattccacacaacatacgagccggaagcataaagtg
taaagcctggggtgcctaatgagtgagctaactcacattaattgcgttgcgctcactgcccgctttccagtcgggaaacctgt
cgtgccagctgcattaatgaatcggccaacgcgcggggagaggcggtttgcgtattgggcgctcttccgcttcctcgctca
ctgactcgctgcgctcggtcgttcggctgcggcgagcggtatcagctcactcaaaggcggtaatacggttatccacagaat
caggggataacgcaggaaagaacatgtgagcaaaaggccagcaaaaggccaggaaccgtaaaaaggccgcgttgctg
gcgtttttccataggctccgcccccctgacgagcatcacaaaaatcgacgctcaagtcagaggtggcgaaacccgacagg
actataaagataccaggcgtttccccctggaagctccctcgtgcgctctcctgttccgaccctgccgcttaccggatacctgt
ccgcctttctcccttcgggaagcgtggcgctttctcatagctcacgctgtaggtatctcagttcggtgtaggtcgttcgctcca
agctgggctgtgtgcacgaaccccccgttcagcccgaccgctgcgccttatccggtaactatcgtcttgagtccaacccggt
aagacacgacttatcgccactggcagcagccactggtaacaggattagcagagcgaggtatgtaggcggtgctacagagt
tcttgaagtggtggcctaactacggctacactagaagaacagtatttggtatctgcgctctgctgaagccagttaccttcgga
aaaagagttggtagctcttgatccggcaaacaaaccaccgctggtagcggtggtttttttgtttgcaagcagcagattacgcg
cagaaaaaaaggatctcaagaagatcctttgatcttttctacggggtctgacgctcagtggaacgaaaactcacgttaaggg
attttggtcatgagattatcaaaaaggatcttcacctagatccttttaaattaaaaatgaagttttaaatcaatctaaagtatatatg
agtaaacttggtctgacagttaccaatgcttaatcagtgaggcacctatctcagcgatctgtctatttcgttcatccatagttgcc
tgactccccgtcgtgtagataactacgatacgggagggcttaccatctggccccagtgctgcaatgataccgcgagagcca
cgctcaccggctccagatttatcagcaataaaccagccagccggaagggccgagcgcagaagtggtcctgcaactttatc
cgcctccatccagtctattaattgttgccgggaagctagagtaagtagttcgccagttaatagtttgcgcaacgttgttgccatt
gctacaggcatcgtggtgtcacgctcgtcgtttggtatggcttcattcagctccggttcccaacgatcaaggcgagttacatg
atcccccatgttgtgcaaaaaagcggttagctccttcggtcctccgatcgttgtcagaagtaagttggccgcagtgttatcact
catggttatggcagcactgcataattctcttactgtcatgccatccgtaagatgcttttctgtgactggtgagtactcaaccaagt
cattctgagaatagtgtatgcggcgaccgagttgctcttgcccggcgtcaatacgggataataccgcgccacatagcagaa
ctttaaaagtgctcatcattggaaaacgttcttcggggcgaaaactctcaaggatcttaccgctgttgagatccagttcgatgt
aacccactcgtgcacccaactgatcttcagcatcttttactttcaccagcgtttctgggtgagcaaaaacaggaaggcaaaat
gccgcaaaaaagggaataagggcgacacggaaatgttgaatactcatactcttcctttttcaatattattgaagcatttatcag
ggttattgtctcatgagcggatacatatttgaatgtatttagaaaataaacaaataggggttccgcgcacatttccccgaaaa

gtgccacctgacgtctaagaaaccattattatcatgacattaacctataaaaataggcgtatcacgaggccctttcgtcgagct
ccataaactttgtttgtacactagacaaacaaatttaaccgcattataatttagttggtttgtatcttacctatgaggaattgaaac
gagaccatcttatcacttgaaattggaagggagattctttattataagaattgtgaaaaaggaggaaaaaaaatgggatccat
gcgtaaaggcgaagagctgttcactggtgtcgtccctattctggtggaactggatggtgatgtcaacggtcataagttttccgt
gcgtggcgagggtgaaggtgacgcaactaatggtaaactgacgctgaagttcatctgtactactggtaaactgccggtacc
ttggccgactctggtaacgacgctgacttatggtgttcagtgctttgctcgttatccggaccatatgaagcagcatgacttcttc
aagtccgccatgccggaaggctatgtgcaggaacgcacgatttcctttaaggatgacggcacgtacaaaacgcgtgcgga
agtgaaatttgaaggcgataccctggtaaaccgcattgagctgaaaggcattgactttaaagaagacggcaatatcctggg
ccataagctggaatacaattttaacagccacaatgtttacatcaccgccgataaacaaaaaaatggcattaaagcgaattttaa
aattcgccacaacgtggaggatggcagcgtgcagctggctgatcactaccagcaaaacactccaatcggtgatggtcctgt
tctgctgccagacaatcactatctgagcacgcaaagcgttctgtctaaagatccgaacgagaaacgcgatcatatggttctg
ctggagttcgtaaccgcagcgggcatcacgcatggtatggatgaactgtacaaatgaggtctcgtttgtatcttacctatgag
gaattgaaaccaggcatcaaataaaacgaaaggctcagtcgaaagactgggcctttcgttttatctgttgtttgtcggtgaac
gctctctactagagtcacactggctcaccttcgggtgggcctttctgcgtttatacaattgtaaggaatattcagcaatttgccc
gtgccgaagaaaggcccacccgtgaaggtgagccagtgagttgattgctacgtaattagttagttagcccttagtgactcgc
atg

Function annotation of pZL02 plasmid:

Pxyla sequence, repeat sequence, xylR sequence, counter-selection marker based on gfp gene for guide RNA insertion, repB sequence, Amp^R sequence, Kan^R, colE ori sequence, Counter-selection marker based on cherry gene for NHEJ insertion.

NHEJ_Bme genes sequence

gtaaaacgacggccagtgaattcggagttaactgcctcgtccattttttgcttaatggaggttgtcatgaaaatgacaaacaa
cgtccaaacaattgccataatcgtttacgcatagtttcgatttcatcgcgtaaaataatttgtgaatgtattcacaataataagaa
gggagaatagtgatgaaaccgatgctgcctactttaacctttgatgtcccaaaaggagatgattggctgtttgaagtgaaatat
gacggtttccgggcgattttggaagtgggcgaaaccgtttcgcttacaagccgcaacggaaaagatcttcttcctctttttccg
gaaattgaacatttttaaaaaactccttggatcacttatcaccttacctgccttgtactcttgacggcgaacttgtttttcttaaaa
atccatacaaagcaaatttcgctgccatccaagttcgcggccgcatgagatctcagcagcgcatcattgaaaaagcacgca
aagctccgtgtcgcctgcttatttttgatgtcttgaaattaaaaggcaaacaagtaagaaatattgactaccaagaaagaaaac
aaatgctgtttgatttttttgaaaaaacaggacttccgttagctccggatgaaaatgatgaaagacttgtacagttcataccggc
aaataacaactttcacaccatttgggaaaacattgtcatttatgacggggaagggattgttgcaaaacagttgaaaagccgct
gggaagaaggaaaacgcacaacaacgtggctgaaatataaaaattggaaatatgtttcttgttttataacggcatatgaaaaa
tcgaacggctatttttacgcagcagtttataaggacaaaaaaatttacccaatcgggcagtttctttttggattaaaacctgatga
aaagcaagcactttttcagatcataaaagagaatatgaccggtgaagacaaacagtttatttacgtcgatccggccatttgcgt
tgaagtgaagtacctggaaatgtatgaggaacaaatgagagaacctcattttaaccgcttccgtttcgagcttttaccggaag
actgtacatatgagaaatttttattacaacagaaaaatttacctgttgaagtagaaattactcatcctgaaaaaccattatgggaa
actccgccgattcaaaagatggactatcttcactacttacgggaaatttccccttatatgcttccatttttaaaggaccgcttattg
acggtaatccgctatccacatggaatttttggcgaagctttttatcaaaaaaattgcccggattatgctccggattttgtcgaca
ctcacgaagaagaaggcatccaatatatcgtatgcaacaatcttaaatcatttttatggcttggaaaccagcttgcctttgaattt
catattcctttccaaacgatcacaagccaaggtccgagcgaaatcgtttttgatttggatccgccatcaaaagatgcatttcctt
tagcaataaaagcggccattaatataaaagaggtgctggaccatctggagctgatcagctttattaagacatccggaaacaa

aggtcttcaagtgtacgtccctttgccggataaccgttattcctatgaggatacaagactgtttacgtcttttgttgccgattatctt
gtttcgaaggatccgggttcctttacaacggaaagaatgaaaaaaaagcgcggaaatcggctgtacgtggactatgtccaa
catgcggaaggcaaaacgatcattgccccgtattcaccgcgcggcaactcaaaagcgactgtagctgctccgttattttggg
aagaagtaaatgagaacctgtcaatagagtcctttcaagtaacctcaattttaaaacgtttgaaagacagcggagatccgtttc
aaactttttttcaaggcaaaagaattgcaaagatttcagcctgttttagatttcttaaaacgcaaatgaggagttaactgcctcgtc
catttttttgcttaatggaggttgtcatgaaaatgacaaacaacgtccaaacaattgccataatcgtttacgcatagtttcgatttc
atcgcgtaaaataatttgtgaatgtattcacaataataagaagggagaatagtgatgcatacaatttggaaaggcagcataag
ctttggacttgtaaatatcccgattaaacttcatgcagcaacagaagacaaggacgttaaactgcgcaaccttcataaagaat
gcaaatcgccaataaaatatgaaaaagtttgtcctgtctgtgatgtggaagtaaaaaatgaagacatcgtaaaggcttacgaa
tacacaaaaggaaaatttgttgttcttgatgaagaagatttggaaaatttgaaacgcgaaaatgaagacaaagcagtggaaat
cattgactttgtaaaaatggaacaaatcgacccgatttactatgacagaagctattatatgtcaccaggtgatggcgggggaa
aagcttattcattactgcgcaaggcgcttgaagaatcggaaaaagtcggcatcgccaaaatcatcatccgctcaaaagagc
agttagccgttatccgggtttatgaaaatacgcttgttatggaaacgatccattatccggatgaagtaagatctgcagccgac
gttccgaatgtgccggcagaaaataaagtaacgaagaaagaaattgatacggccattttattaattgaccagctgacaacgg
attttcaaccagaaaaatatacggatgactaccggacggcattacttgaattaattgaagcgaaacgtacaggaaaagaagt
cgtgacaccggcagaaaaagaacctgtttcgaatgtaacggatttaatggctgcattgcaggcttccattgaccgcacaaaa
ccgaaaaagacggaaacacgcaaaaaacgcactgccacaaaaacaaagagagaagcgtaacttaag

Annotation of NHEJ_Bme genes:

P_{ldh} promoter, LigD_Bme, Ku_Bme

NHEJ_Bth genes sequence

gtaaaacgacggccagtgaattcggagttaactgcctcgtccatttttttgcttaatggaggttgtcatgaaaatgacaaacaa
cgtccaaacaattgccataatcgtttacgcatagtttcgatttcatcgcgtaaaataatttgtgaatgtattcacaataataagaa
gggagaatagtgatgataccagctgttcaaagctcttttattaaaaaacgggtgataaaaatgaaaccaatgctgcctacatta
tcctttgatgttccaaaagaagaaaaatgggtttatgaaattaagtatgacgggtttcgtgccatcatacaaatggatgatcaa
ggaattgatatgattagccgcaacggtaaaagcctgattgaccagtttcctgaagcagtcaattttttaatcattataaaaaac
aattatccgatttttttccgatcttattggacggtgaattgaccattttggaaaatgacagcaagagcgatttttttcgtttgcagac
tcgcggacgcatgcgcagcaaaaagcgcatttttgaagcatcgctgtcaaacacttccacttatcttgcgtttgacttacttca
attaaaaggtcaatgggtgacaaagcagccttttttaaaacgcaaggaactgctttatcaagtgatgaagcattgcagccttc
cgctttcaccggacccggccaatcattgctttatccagtttcttccatttcataaagaatttcatgaagtttgggaaacggtcaag
cagaaagaaggagaaggaatcgtcgcaaagcaggcagaaagcaaatggattgaaggaaagagaacgacacaatggtt
aaagatcaaaaattggaaaaaagtgcactgtttcattactgcttttgaaaaaaacaacggatttttttcatgttgctgtttatgaaaa
agaaaagatcattccgataggtcttttcaaaaatggaatgaatcaggatgaaacaaatatattgattcaaataatgaagaaaaa
tgccaccgatgaagacgcccaattcgtgtatgtacagccttcaatatgtgtagaattattttatttacaattttatgatggaacttta
cgtgaaccgtttttttctcaatttcttttccagacatcaccggctgactgtaccctttccaagatgatggaaatgtccgaagtgaa

caagacggaaactgatacggatattccgatcaatatcacccatccagaaaaaccgctttgggaaaaaaattcaataacgaa
attagattatattaaatatctgaaatttattttcccttatatggaaccgtttttaagaaatcgcttacttactgttatccgctatccgca
tgggattttcggggaagctttttttcagaaaaactgtccggactatgcaccggactttgtcgaaacttatttggacgaaggaat
aaattatattttatgcaataattttgaaacatttctttggttaggcaaccagttggcaattgaatttcataccccattccgcaccatt
gataaaaaattcccgacagaagttgttattgatttagatccgccaacaaaggcagaatttccgctggccatcgaagctgccct
ttatttaaaggaagatattattgacaagttaggacttcatgcttttgtcaaagtgtccggcaatcggggcattcaagtgtattttcc
gttatccggcaaccgcatcacttggaatcaaacgcgcaagttcactgaatttatcgccaattatttacttatgaaaaatgacaa
gcattttacaattgaacggttaaagaaaaaccgcggtcatcggttgtatgttgattacatccaacatgcaaaaggaaagacga
tcatttgcccgttttcggttcggggaaatgcaaacgccggtgtggccgcaccgctggaatgggatgaattaaccgataacat
caccccggacggtttttccatagatgtggttatgaaacgcatagaaggaaaaggaaatccatttgcttctttttttcaagttgac
aatgacgggccattacaggaaatgattcgtttcataaaagacaagtaaggagttaactgcctcgtccatttttttgcttaatgga
ggttgtcatgaaaatgacaaacaacgtccaaacaattgccataatcgtttacgcatagtttcgatttcatcgcgtaaaataattt
gtgaatgtattcacaataataagaagggagaatagtgatgcatacaatgtggaaaggaagcatcagcttcgggttagtaaat
attccggtaaaattgcatgctgcgacggaagataaagatgtaaaatttcggagcctgcataaaaaatgtcataccccgatca
aatatcaaaaagtatgtccgaattgtaatatagaaatctccaatgatgaaattgtgcgcgcttatgagtatacaaaaggaaaat
atatcgttattgaggatgaagattttgccggccttgaaaaagaaatagaagataaagcagttgagattatcgattttgtgaaaat
cacagatattgatccgatttattttaaccggagctactatttgtctccgaatgaaggaggggttaaagcgtattcgttattaagaa
aagcgttgcaagaaactgaaaaagtcggtattgccaaaattgtcatccgctccaaagagcagcttgccgtgatccgggttta
tgaaaacacattggtaatggagacaattcattatccggatgaggttcgcaaagcaaaggacgtaccgaatgtcccttctgcta
accaaataacaaagaaggaattggacacggccattcttctgattgatcaattatcaacggaatttcaaccggaaaaatatcat
gatgaatatcgcgaagcggtattaaacttagttgaagcaaaacggacaggagaagaaatggtgacaccggagacaaaag
ataagccgaataatgttatggatttaatggcagcattacaagcttccatagaccgcacaaaaccgaatcgggaaacaaaag
aaaaaccggtaaaaaaacggaaacctgttacccgtgccaagaaaaaagcttag

Annotation of NHEJ_Bth genes:

P_{ldh} promoter, LigD_Bth, Ku_Bth

Supplementary Note 2

This code is used to generate guide RNA for a spectrum of long-range genomic deletions in *Parageobacillus thermoglucosidasius.*

```
import re
datafin = []
with open(r'GCA_001700985.1_ASM170098v1_cds_from_genomic.txt') as
file_object:
    data1 = file_object.read()
    data2 = data1.split('>')
    while ''in data2:
        data2.remove('')
    for data in data2:
        p = data.replace('\n', '')
        if len(p) > 0:
            datamid = []
            i = p.rindex(']')
            datafin.append('>')
            datafin.append(p[:i+1])
            datafin.append('\n')
            c = p[i + 1:]
            basepair = {'A': 'T', 'T': 'A', 'G': 'C', 'C': 'G'}
            revc = []
            for i in c:
                revc.append(basepair[i])
            revc = ''.join(revc[::-1])
            m = len(c) - 38
            x = 'TTA'
            q = [x.start() for x in re.finditer(x, c[0:m])]
            q0 = [x.start() for x in re.finditer(x, revc[0:m])]
            n = len(q)
            revn=len(q0)
            #if n > 4:
            for i in q:
                r = c[i + 3:i + 41]
                if r.count('A')+r.count('T')>19:
                    datamid.append(r)
            for i in q0:
                r = revc[i + 3:i + 41]
                if r.count('A') + r.count('T') > 19:
                    datamid.append(r)
```

```
            def order(elem):
                return elem.count('A')+elem.count('T')
            datamid.sort(key=order,reverse=True)
            if len(datamid)>3:
                datamid=datamid[:3]
            for data in datamid:
                datafin.append(data)
                datafin.append('\n')
f2 = ('a spectrum deletions.txt')
with open(f2, 'w')as file_object:
    file_object.writelines(datafin)
print('fine!')
```
